# Masked Face Analysis via Multi-Task Deep Learning

**DOI:** 10.3390/jimaging7100204

**Published:** 2021-10-05

**Authors:** Vatsa S. Patel, Zhongliang Nie, Trung-Nghia Le, Tam V. Nguyen

**Affiliations:** 1 Department of Computer Science, University of Dayton, Dayton, OH 45469, USA; patelv20@udayton.edu (V.S.P.); niez01@163.com (Z.N.); 2 National Institute of Informatics, Tokyo 101-8430, Japan; ltnghia@nii.ac.jp

**Keywords:** multi-task learning, masked face, age, gender, expression, face detection

## Abstract

Face recognition with wearable items has been a challenging task in computer vision and involves the problem of identifying humans wearing a face mask. Masked face analysis via multi-task learning could effectively improve performance in many fields of face analysis. In this paper, we propose a unified framework for predicting the age, gender, and emotions of people wearing face masks. We first construct FGNET-MASK, a masked face dataset for the problem. Then, we propose a multi-task deep learning model to tackle the problem. In particular, the multi-task deep learning model takes the data as inputs and shares their weight to yield predictions of age, expression, and gender for the masked face. Through extensive experiments, the proposed framework has been found to provide a better performance than other existing methods.

## 1. Introduction

**Figure 1:**
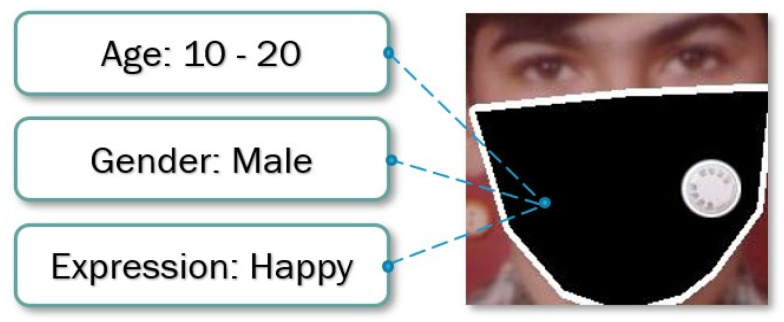
The exemplary input and output of the proposed method. The age, the gender, and the expression are predicted from the given masked face.

Face recognition has been one of the active research problems studied in computer vision for decades due to its many practical applications, for instance, in the automotive industry, security, retail, beautification, and social networks [1,2,3,4,5]. So-called facial expression recognition systems are computer programs which aim to automatically translate and understand facial actions from visual information. The processing of facial expressions is often confused with emotional interpretation in the field of machine vision. Due to the high demand for facial expression recognition systems, there have been many developments in this field. Due to the COVID-19 pandemic, which has caused many people to wear face masks to prevent infection, it has become urgent to meet the challenge of analyzing faces wearing masks. There are few methods that have been introduced to create a face mask dataset [6], detect the face [7], recognize facial identities [8,9], enable multitask learning [10,11,12], and recognize facial features [13]. The face detection systems have been developing over the years but due to COVID-19 everything has come to a hold [14], as the wearing of masks has meant that earlier methods have struggled to analyze human faces. The idea proposed here will overcome this problem, the study assisting not only in predicting the age of a person wearing a face mask [1,15,16] but also in predicting gender [2,17,18] and mood (expression) [19,20]. Moreover, we will also release our masked face dataset upon publication. Multitask learning with different backbones gave better results for our created dataset than other methods. The input and the output of the method are shown in Figure 1.

The main contributions of this paper are three-fold. First, we introduce the simple yet effective mask synthesis method. Second, we build the dataset of masked faces with three separate modalities (i.e., age, gender, and expression). Third, we propose the multi-task deep learning framework to tackle the problem of face recognition. Finally, we conduct experiments on the multitask learning model and compare it with the single models [12,21]. To make this possible, we need to have a good dataset with appropriate labels as an input, which is not available in the current market, so we have introduced the face mask dataset. The dataset of face with labelled ages is derived from FG-NET [16]. After rendering the faux mask on the faces, we manually added labels of gender and expression [6]. To this end, landmark points on the face were generated using the generated landmark points extracted from a landmark point detector [22]. Following the dataset collection, we evaluated different separate models for each label (age, gender, and expression), such as the Local Binary Pattern (LBP) [23], Eigenfaces with Support Vector Machine (SVM) classifiers [17,18,20], deep learning models with two backbones—traditional Convolutional Neural Networks (CNN) [21] and Residual Neural Network (ResNet)—and compared the performance. Finally, multitask deep learning [10,11,12] was evaluated, and was found to outperform single task learning by reducing the effort of constructing different models for each task and yielding more accurate results.

The rest of the paper is organized as follows. The related works are summarized in Section 2. The dataset and the computational framework are introduced in Section 3. Section 4 presents the experimental results. Finally, Section 5 concludes the paper and paves the way for future work. 

## 2. Related Work

This section explores current facial datasets. Then, we go through the early studies on facial recognition [24,25], used for feature classification, as well as the various techniques for identifying the face. Finally, we discuss mask face analysis briefly in order to analyze existing work on facial identification with various backbones.

### 2.1. Face Datasets

Many previous research studies, such as FG-NET [26], LFW (Labelled Faces in the Wild) [27], and Yamaha [28], among others, have developed databases for facial recognition [8,9] that are being used in a variety of research projects. The Yamaha dataset [28] only includes Asian faces with no annotation, the large-scale LFW dataset [27] lacks annotation, and the FG-NET dataset [26] contains 926 images including human age annotation. As a result, we are using the FG-NET dataset for our system. 

There have been several previous studies that have generated datasets and conducted various tasks using them. Wang et al. [6] proposed three separate forms of datasets to recognize individuals wearing masks, including the Masked Face Detection Dataset (MFDD), the Real-world Masked Face Recognition Dataset (RMFR), and the Simulated Masked Face Recognition Dataset (SMFR). Similarly, many approaches have used datasets and incorporated them into their frameworks, but none of them met our requirements. Consequently, in our scheme, we use FG-NET [26] as the base and further annotate gender and expression and apply masks to the photos of faces to construct our dataset.

### 2.2. Face Recognition

For face recognition, the crucial step is to extract facial features known as “signatures”. There are several methods for extracting the shape of the lips, eyes, or nose to classify the face based on its scale and distance. Some techniques that are widely used to extract these facial features, such as the Histograms Oriented Gradient (HOG) [29,30] and Eigenfaces, have shown good performance in terms of system speed and accuracy. Since the Eigenfaces method is primarily a dimension reduction method, a system can represent a large number of subjects with a small amount of data. There are other techniques available, such as Independent Component Analysis (ICA), Scale-Invariant Feature Transform (SIFT) [31], the Gabor filter, Local Phase Quantization (LPQ), Haar, and the Local Binary Pattern (LBP) [32,33]. Here, the LBP is a basic but effective textural feature that marks pixels in an image by thresholding each pixel’s neighborhood and treating the result as a binary number. Principal Component Analysis (PCA) [34,35], which is used in multiple applications and has a variety of outcomes, was implemented into our dataset to generate the predicted labels. We can derive a wide variety of features from images using CNNs. This feature-extraction concept can also be applied to face recognition. For example, in a binary classification, where two images of the same person are passed in, the network should return identical outputs (i.e., closer numbers) for both images; while images of two different people are passed in, the network should return somewhat different outputs for both images. The CNN is used to extract the most important data characteristics of the faces, and then the k-nearest neighbor (K-NN) is utilized as a classifier. As the predictive utility of a strong instance value, the K-NN algorithm employs neighborhood classification. An instance-based learning with K-NN [36] is widely used in many applications. In [5], Adjabi et al. reviewed facial recognition in both 2D and 3D images. Ulrich et al. [37] analyzed the use of RGB-D images for supporting different facial usage scenarios. Bock et al. [38] explored the use of low-cost 3D cameras in security. Likewise, Ruiqin et al. [39] introduced a face recognition access entrance guard system. Dagnes et al. [40] investigated face recognition with eye and mouth occlusions in 3D geometry. 

There are few methods that have been introduced for emotion recognition [19,20], gender recognition [2,17,18], and age prediction [1,15,16], performing separate tasks for each. There are many methods implementing multiple tasks with separate models, which is not always feasible. As an effort of incorporating multi-task learning, Vandenhende et al. [12] review papers on multitasking and variants such as hard parameter sharing, soft parameter sharing, Encoder-focused models and Decoder-focused models. In our framework we are focusing on hard parameter sharing and sharing data on age, gender, and expression in such a way. 

### 2.3. Masked Face Analysis

Many prior works have focused on facial recognition in cases of occlusion [41,42]. The work has been conducted in a number of ways, including identification of the face in the wild, twin recognition [43], occluded face detection [41,42], detecting the face between the mask and the actual face, and the use of Generative Adversarial Networks (GANs) for face modulation and detection. There are also a few studies of detecting faces with masks.

To detect masked faces in the wild, Ge et al. [9] created a dataset dubbed MAFA. Then, they proposed Locally Linear Embedding CNN (LLE-CNN) method with three modules. The proposal module first combines two pre-trained CNNs to extract candidate facial regions from the input image. Then, the embedding module turns feature descriptors into vectors of weights with respect to the components in pre-trained dictionaries of representative normal faces and non-faces by using locally linear embedding. The verification module takes the weight vectors as input and identifies real facial regions, as well as their accurate positions, by jointly performing the classification and regression tasks within unified CNNs. There also exist research efforts [44] to detect the identity of a person with a face mask or without a face mask using OpenCV and the Haar Cascade.

We note that the single-model based research on human face recognition has recently achieved state-of-the-art results. However, there are few examples of research into facial identification with masks achieving high accuracy results when it comes to recognizing faces [36]. In this work, rather than using a single model for each task, we aim to simultaneously train multi-task for predicting age, gender, and expression.

## 3. Data Collection and the Proposed Framework

**Figure 2:**
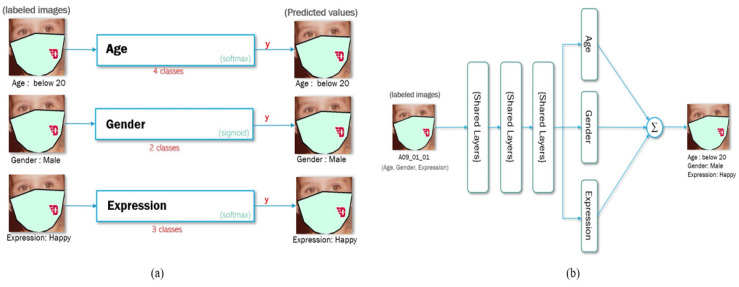
Visualization of deep neural network of (**a**) a single model with single input and output of individual models and (**b**) a multitask neural network with single input and single output with multiple labels.

In this section, we introduce the FGNET-MASK dataset and the multi-task deep learning model. The two different methods, namely, single and multi-task learning, are shown in Figure 2.

### 3.1. FGNET-MASK Dataset Collection

**Figure 3:**
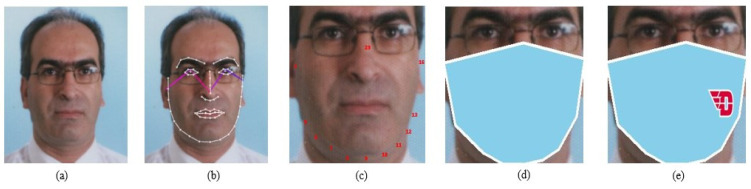
The flowchart of our masked face synthesis. (**a**) is the original image from the FGNET dataset. (**b**) is the image rendered with 69 facial landmark key points. (**c**) shows the landmark points selected to create the mask. (**d**) shows the mask rendered and the face cropped by taking minimum and maximum values. (**e**) shows the external logo embedded.

The most important step in the framework is the creation of the dataset. It is extremely difficult to assemble a dataset of individuals of various ages, genders, and expressions wearing masks, so we rendered the mask and labelled the images. The construction of the FGNET-MASK dataset is detailed as follows (cf. Figure 3). 

**Figure 4:**
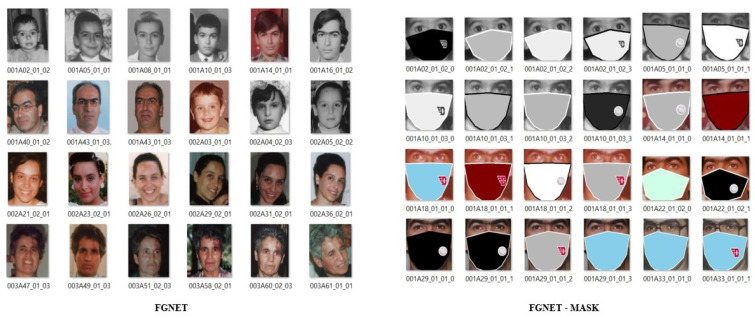
(**Left**) The original images from FGNET. (**Right**) The synthesized images of our FGNET-MASK dataset.

First, the human face images (without a mask) from FGNET [26] were adopted with their previously labelled ages. Then, the dataset was further manually labelled with the individuals’ gender and expression. In total, we obtained 925 images with three types of labels on each image. Next, the images were run through OpenPose [22] to detect and generate 2D landmark points on detected faces in the dataset. Eventually, we synthesized the face mask using the Pillow package [45], after receiving the landmark points, with a variety of colors and logos. Since the initial dataset only contains 925 images, which is insufficient for training a machine, we constructed four replicas of each masked image with various permutations of mask color, as shown in Figure 4. We also changed the undetected landmark points, resulting in the final FGNET-MASK dataset of 3404 images with rendered face masks, which is sufficient for a machine to be trained. Age, gender, and expression were all branded in the dataset. The dataset contains four age categories: under 10, 10–20, 20–40, and over 40 to balance the samples for each age group. There are only two genders labelled: Male and Female. Finally, expression labels were classified as Happy, Neutral, or Unhappy. 

Finally, the FGNET-MASK dataset is fully annotated. The total number of pictures grouped and categorized into their age groups is 1400 images of individuals under the age of 10, 1010 between the ages of 10 and 20, 720 between the ages of 20 and 40, and 274 over the age of 40. For the two gender categories, Male and Female, there are 2000 and 1404 images, respectively. And for the three types of expressions, there are 1800 images with happy expressions, 950 with neutral expressions, and 654 with unhappy expressions. The outcome of the FGNET-MASK dataset is shown in Figure 4.

### 3.2. Single Models

Following the creation of the FGNET-MASK dataset, which included rendering the mask and labeling the images, the images were fed into three distinct CNNs for Age, Expression, and Gender. The model ‘Age’ is a multi-class classification with four distinct classes based on criteria of less than 20, 20–30, 30–40, and greater than 40. The ‘Expression’ model is also a multi-class classification model with three classes: happy, neutral, and unhappy. The final model, ‘Gender,’ is a binary classification with Male and Female options. 

According to the categories of the respective classes, the single model has three convolutional layers, in which each is followed by a maximum pooling layer, and dense layers. Convolutional layers use a filter to make a feature map that summarizes the presence of detected features in the input. Maximum pooling layers are expected to downsample feature detection in feature maps. We used Adam optimization for training deep networks. Our newly constructed dataset was tested again with a different network with higher complexity of convolutional layers and maximum pooling, namely, ResNet152, with 60,430,849 total parameters [45], to compare its accuracy with the traditional CNN model with only 7654 parameters. ResNet-152 [46] used pre-trained weights on ImageNet as their weights to train the model. The top fully connected layers were excluded, and the model was fine-tuned with 137 layers out of 152.

For the single model, we considered using LBP and Eigenfaces with an SVM classifier. The Local Binary Pattern [16,32,33] is a simple texture operator that marks the pixels of an image by thresholding the neighborhood of each pixel and treating the result as a binary number. After pre-processing, the dataset was transformed into decimal numbers and fed into the SVM model using a linear kernel. All the data linearly separated using this kernel were used to separate models for age, gender, and expression, and the results were reported, deep learning outperforming the LBP-SVM process. Following the LBP implementation, the dataset was further implemented with Eigenfaces using PCA on SVM models, but the results were worse than those obtained with the LBP.

### 3.3. Multi-Task Deep Learning

Multi-task deep learning (MTDL) is an inductive transfer learning approach that involves the cooperative training of two or more learning machines. MTDL refers to the mechanism by which a machine learns as it moves from one task to the next. The idea is that each task should benefit from the knowledge gained while preparing for other related assignments. Deep multi-task architectures were divided into two types: hard parameter sharing techniques and soft parameter sharing techniques. The parameter set is split into shared and task-specific parameters in hard parameter sharing. In this proposed method we are using the hard sharing parameter. MTDL models using hard parameter sharing typically consist of a shared encoder that branches out into task-specific heads.

The most common hard parameter sharing design includes a shared encoder that branches out into task-specific decoding heads. Backpropagation in MTDL is the most efficient method for solving learning distributed representations. For example, in every model, the equation will be the same, if *M* > 2 (i.e., multiclass classification). We calculated a separate loss for each class label per observation and summed the result. For example, $L_{age}$, the loss function of the age model was computed as:

(1) $$L_{age}=-\overset{M}{\underset{c=1}{\sum }}y_{o,c}log\left(p_{o,c}\right)\;$$

where M is the number of classes (below 10, 10–20, 20–40, and 40 and above, *y* is the groundtruth label, *p* is the predicted probability that observation *o* is of class c. Meanwhile, the total loss function L for the multitask model was computed as follows:

(2) $$L=L_{age}+L_{gender}+L{\;}_{expression}$$

The total loss function here solves optimization problems at the same time: minimization of loss function and making a normalization of our parameters. Our proposed multi-task learning followed this approach. Following the sharing of the layers with the data, the output was determined in accordance with the specified task (i.e., age with respect to gender and expression, gender with respect to age and expression, or expression with respect to age and gender).

## 4. Experimental Results

In this section, we compare the proposed method for masked face analysis with two implementation variants: basic CNN and ResNet-152. We also compare the single model with the multitask learning model. We included many baselines in the evaluation, such as EigenFace [30], LBP [23], TinyImage [47], and VGG Face [48]. All experiments were conducted on the testing set of the collected FGNET-MASK dataset. Regarding the results, we adopted accuracy as the main performance metrics:

(3) $$Accuracy=\frac{\sum_{i=1}^{k}\frac{tp_{i}\;+\;tn_{i}}{tp_{i}\;+\;tn_{i}\;+\;fp_{i}\;+\;fn_{i}}}{k}$$

where $tp_{i}$, $tn_{i}$, $fp_{i}$, $fn_{i}$ are the true positive, the true negative, the false positive, and the false negative, respectively. Meanwhile, k is the number of classes for each classification task. 

### 4.1. Single Model

Three distinct models were developed in the deep learning system by using two separate backbones (simple CNN and ResNet-152) and three distinct approaches were also used in the SVM method. The results of the model predicting age, gender, and expression were phenomenal with the deep learning methods compared to those with the models using the SVM method (the LBP and SVM). The testing precision of the single trained models is as follows.

#### 4.1.1. Support Vector Machine (SVM)

The Support Vector Machine (SVM) is a supervised machine learning algorithm that can be used to solve classification and regression problems. In the SVM algorithm, each data object is plotted as a point in *n*-dimensional space (where *n* is the number of features), with the value of each element being the value of a certain coordinate. Then, classification is performed by finding the hyper-plane that differentiates the two or more classes according to the requirement. We use linear SVM as the classifier for LBP, Eigenfaces, TinyImage, and Multi-Block Color-Binarized Statistical Image Features (MB-C-BSIF). As shown in Table 1, the LBP gives an unsatisfactory performance. Using the same process, we have implemented and compared our results for Eigenfaces obtained from PCA (Principal Control Analysis) [2], which is the method of calculating the principal components and using them to modify the basis of the data. The results with Eigenfaces are slightly better than those obtained with the LBP. Regarding TinyImage, the face image is downsized into 32 × 32, and the features are extracted by concatenating all image pixels. The extracted TinyImage features are used to train an SVM model, yielding results that are better than Eigenfaces, the LBP, and single task CNN. For MB-C-BSIF [49], the extracted features do not perform well, i.e., on par with LBP. One possible reason is that MB-C-BSIF possesses a large dimensionality. That may cause overfitting in the model training. Meanwhile, the features extracted from the VGG Face [48] using a pretrained model, on the other hand, outperform all other feature types. 

#### 4.1.2. Simple Convolutional Neural Network (CNN)

**Figure 5:**
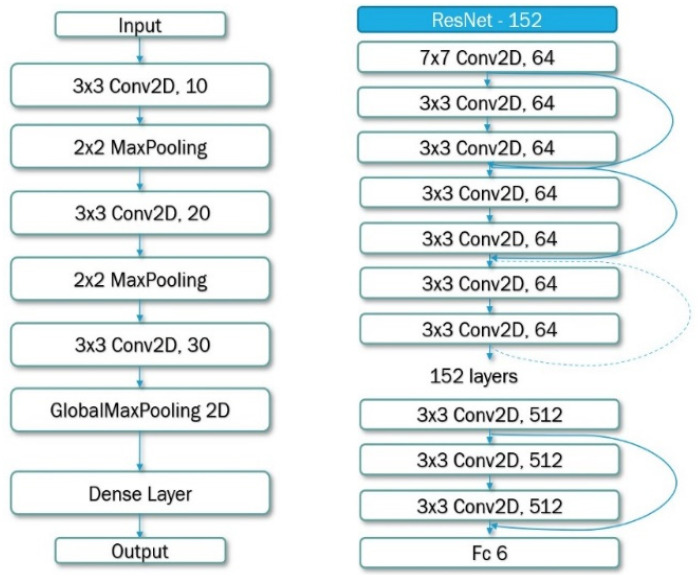
Backbones used in our implementation: (**left**) simple CNN; (**right**) ResNet-152.

In this work, we first tried a simple CNN model (as shown in Figure 5, left) for each class, namely, age, gender, and expression. Each model was trained using the same CNN architecture but with different activation functions for binary and multiclass classification. The age model’s accuracy on unknown testing data was 0.68, the gender model’s testing accuracy was 0.77, and the expression model’s accuracy was 0.60. 

#### 4.1.3. ResNet-152

Furthermore, we tried a deeper network, namely, ResNet-152 (as shown in Figure 5, right). By using the deeper model, the result of the age classification task reached 0.91, whereas gender and expression classification results obtained 0.95 and 0.82, respectively. Here, the accuracy rate obtained from the residual neural network-ResNet-152 was significantly higher than that obtained from the other approaches we used, SVM and Deep Learning (CNN). 

### 4.2. Multitask Deep Learning Model

**Figure 6:**
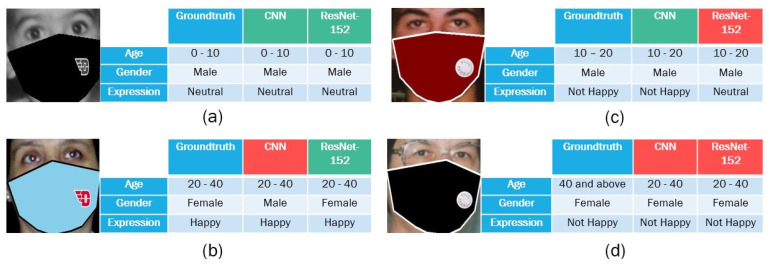
The exemplary pictures of age, gender, and expression prediction of an MTDL model from both backbones (CNN and ResNet). Green indicates that the expected values match the ground-truth; red indicates that they do not.

The method of designing multiple models for multiple labels was exhausting and unconvincing, so the concept of using multitask deep learning was a brilliant way to save time and effort by creating just one model for the requisite multiple labels. The MTDL technique is the best approach to getting better results when compared to single CNN models. Results obtained after comparing the model are far conversing, with respect to age, gender, and expression. Figure 6 showcases the example results. Regarding the simple CNN, the testing accuracy obtained using the CNN backbone for each class was better than the single models. The testing precision obtained after conducting the multitask with respect to age, gender, and expression was 0.74, 0.83, and 0.70, respectively. This evidently outperforms the single models in terms of output. Meanwhile, the ResNet-152 model produces a better performance than the simple CNN model. In particular, the results for age, gender, and expression were 0.95, 0.98, and 0.9, respectively. This clearly demonstrates that the deeper backbone tends to obtain the better performance in multi-task deep learning. It should be noted that our work can be adopted in many contexts, such as surveillance systems, person re-identification, targeted advertisement, to name a few.

## 5. Conclusions and Future Work

In this paper, we investigated the problem of human masked face recognition. We constructed FGNET-MASK, a new masked face dataset with different modalities via face synthesis. We then proposed a multi-task deep learning (MTDL) method to give a prediction of the age, expression, and gender of a person wearing a face mask. The experiments show the impressive performance of the proposed method on the testing data. In the future, we would like to collect more data for diversity, and we will also work on different datasets, like RMFRD [6]. In addition, we will investigate different tasks in masked face analysis, such as facial landmark point detection and mask removal.

## Figures and Tables

**Table 1:** Testing accuracy of the models with different backbones.

Accuracy	Age	Gender	Expression
Method			
Eigenface	0.59	0.68	0.58
LBP [18]	0.53	0.64	0.55
TinyImage [47]	0.73	0.82	0.70
VGG Face [48]	0.84	0.89	0.75
MB-C-BSIF [49]	0.48	0.64	0.53
Single task (simple CNN)	0.68	0.77	0.60
Single task (ResNet)	0.91	0.95	0.82
MTDL (simple CNN)	0.74	0.83	0.70
MTDL (ResNet)	0.95	0.98	0.90

## Data Availability

The data presented in this study are available on request from the corresponding author.
